# Neural correlates of children with avoidant restrictive food intake disorder symptoms: large‐scale neuroanatomical analysis of a paediatric population

**DOI:** 10.1111/jcpp.14086

**Published:** 2024-12-02

**Authors:** Michelle Sader, Holly A. Harris, Gordon D. Waiter, Pauline W. Jansen, Justin H.G. Williams, Tonya White

**Affiliations:** ^1^ University of Aberdeen Aberdeen UK; ^2^ Erasmus MC University Medical Centre Rotterdam Rotterdam The Netherlands; ^3^ Erasmus University Rotterdam Rotterdam The Netherlands; ^4^ Griffith University Gold Coast Queensland Australia; ^5^ Gold Coast Mental Health and Specialist Services Gold Coast Queensland Australia; ^6^ Erasmus Medical Centre Rotterdam Rotterdam The Netherlands; ^7^ Section on Social and Cognitive Developmental Neuroscience, National Institute of Mental Health Bethesda Maryland USA

**Keywords:** ARFID, eating disorders, MRI, neuroanatomy, BMI, hunger awareness

## Abstract

**Background:**

Avoidant restrictive food intake disorder (ARFID) is a recently recognised feeding and eating disorder and is characterised by a lack of interest and motivation to eat. Despite burgeoning research, few studies to date have explored the underlying neurobiology of ARFID. Research examining the neural underpinnings of ARFID can greatly assist in understanding different mechanisms that play disorder‐specific roles.

**Methods:**

We studied a total of 1,977 10‐year‐old participants from the Generation R Study, a population‐based Dutch cohort, to cross‐sectionally examine neuroanatomical differences between those with versus without ARFID‐like symptoms. Children were classified with versus without ARFID symptoms using the ARFID Index, a validated evaluative tool comprised of parent‐reported and researcher‐assessed measurements of picky eating, energy intake, diet quality, growth and psychosocial impact to characterise ARFID symptoms in the paediatric population. Global and regional values of surface area, cortical thickness, and volume from T_1_‐weighted structural magnetic resonance imaging (MRI) scans in those with ARFID symptoms were compared with children not exhibiting symptoms.

**Results:**

We identified 121 (6.1%) individuals with ARFID symptoms relative to 1,865 (93.9%) individuals without ARFID symptoms. Neuroanatomical findings identified significantly greater frontal (*p* = .00743; *d* = 0.21) and superior frontal (*p* = 6.56E‐04; *d* = 0.28) cortical thickness among children with ARFID symptoms.

**Conclusions:**

This first large‐scale study of the neural correlates of ARFID identified greater thickness of frontal cortical regions in children with ARFID symptoms, suggesting a role for executive function in the aetiology of the condition.

## Introduction

### An overview of ARFID

Avoidant restrictive food intake disorder (ARFID) is a feeding and eating disorder (FED) recently introduced to the Diagnostic and Statistical Manual of Mental Disorders (DSM‐5) in 2013 (American Psychiatric Association [APA], 2013; 2022). ARFID presents as selective and restrictive eating behaviours that hinder to meet nutritional needs and can occur at any age. ARFID often includes psychosocial impairments involving difficulties sustaining relationships and participating in social events (APA, 2022; Iron‐Segev et al., 2020). Additional diagnostic criteria include that the eating disturbance is not: (1) Due to lack of food availability/cultural practice; (2) Attributed to other feeding and/or eating disorders (ED) (i.e. bulimia nervosa, anorexia nervosa) or driven by body shape motivations; (3) Attributed to another mental disorder (e.g. anxiety, depression, obsessive‐compulsive disorder). ARFID is reported as clinically distinct from other FEDs, with three existing symptom profiles encompassing ARFID‐limited intake, ARFID‐limited variety, and ARFID‐aversive/avoidant, in which individuals restrict intake due to low appetite/lack of interest, sensory difficulties/appetitive rigidity, or fear of aversive consequences, respectively (APA, 2022; Norris et al., 2018).

### Existing neuroimaging research on ARFID

Existing neuroanatomical research on ARFID is limited. To date, there are no large‐scale studies examining brain morphology associated with the condition. Only two task‐based functional magnetic resonance imaging (fMRI) studies have been conducted involving ARFID populations, both using food cue processing paradigms evaluating brain response to high‐ versus low‐calorie foods (Getachew et al., 2021; Kerem et al., 2022). Kerem et al. (2022) reported that youth (mean age: 16.9 years) with ARFID with an overweight/obese body mass index (BMI) state (*n* = 11) had significant hyperactivation in the orbitofrontal cortex (OFC) and anterior insula relative to those with ARFID falling within a healthy BMI range (*n* = 12). Getachew et al. (2021) evaluated sex‐based fMRI differences in those with ARFID, reporting no differences (mean age: 16.1 years; *n* = 62). However, neither study included typically developing cohorts to provide case–control comparisons, limiting inferences that can be made on ARFID‐specific neural correlates. Two existing case reports of individuals diagnosed with ARFID have magnetic resonance imaging (MRI) findings of a pineal germinoma (McDonald, Lin, & Bursztyn, 2022) and atrophy of the spinal cord (Chandran, Anderson, Kennedy, Kohn, & Clarke, 2015), but such findings do not necessarily reflect biomarkers or neurocorrelates that could be associated with an ARFID diagnosis.

Despite limited ARFID‐related imaging literature, earlier work in the Generation R Study examining brain structure across the body mass index (BMI)‐spectrum demonstrated an inverted‐U‐shaped relationship between total brain volume and BMI (Steegers et al., 2021). Steegers et al. (2021) found that both higher and lower body weight were associated with lower brain volumes in 3,160 9–11‐year‐old children (White et al., 2018). The BMI‐cortical gyrification association showed an inverted‐U‐shaped curve, in which children with both low and high BMI had decreased gyrification. Since Steegers et al. (2021) evaluated children with low BMI irrespective of the underlying cause, low‐BMI states such as those previously reported in ARFID‐like states (Sader et al., 2023; Sader, Waiter, & Williams, 2023), or FED diagnoses (Murray et al., 2022), may be associated with structural alterations of the brain.

### Potential neuroanatomical regions associated with ARFID

In tandem with existing fMRI studies implicating the OFC and insula, previous theoretical papers examining the aetiological and potential neurobiological origins of ARFID shed light on potential regions of interest associated with the FED. Investigations of potential neurobiological underpinnings associated with ARFID identify differing phenotypes and brain regions implicated in various presentations of symptomatology (Thomas et al., 2017). The authors hypothesised that brain regions central to processing appetite, such as the hypothalamus and anterior insula are associated with the lack of interest ARFID profile, while regions associated with motivational defence and response, such as the anterior cingulate cortex (ACC) and ventral prefrontal cortex (which contain the insula and OFC), are associated with the aversive ARFID profile (Thomas et al., 2017).

Existing neuroanatomical literature in other EDs characterised by dietary restriction, such as anorexia nervosa (AN), may provide insights into possible structural differences present in those with ARFID. A previous systematic review of neural correlates associated with AN identified larger volumes of the ACC, OFC, and superior/middle frontal gyri (Sader, Williams, & Waiter, 2022) relative to controls. Outside of the previously hypothesised insula, ACC and OFC, the superior/middle frontal gyri have also been shown to be associated with volitional appetite control (Althubeati et al., 2022; Devoto et al., 2018) and thus may also be associated with ARFID symptomatology. Such associations may exist due to similarities between ARFID and AN concerning both severity of malnutrition (Alberts et al., 2020), and challenges regarding disorder treatment/outcome, highlighted via reported similarities in rates of recovery or diagnostic persistence (Lange, Ekedahl Fjertorp, Holmer, Wijk, & Wallin, 2019). Specifically, the superior frontal gyrus may be associated with symptomatology relating to the ARFID‐aversive profile through its dense anatomical connections to the ACC (Li et al., 2013). In addition, the superior frontal gyrus has been shown to be associated with tasks involving conflict anticipation (Berkman, Falk, & Lieberman, 2012), such as what would be seen with aversive responses to food. The middle frontal gyrus is reported to serve the spatial orientation of attentional resources (Japee, Holiday, Satyshur, Mukai, & Ungerleider, 2015) as well as context‐dependent goal representations (Turnbull et al., 2019) and is possibly associated with symptomatology relating to both the lack of interest and aversive ARFID profiles.

While accounting for 10% of the total brain weight, the cerebellum contains over 50% of neurons in the human brain (Silver, 2018). Thus, it is not surprising that the cerebellum may also be associated with ARFID symptomatology, notably through its proposed role in appetite. The cerebellum is not only associated with gut‐brain communication governing physiological gastric rhythm via peristalsis (Choe et al., 2021), but is also associated with EDs and BMI. Cerebellar dysfunction is implicated in multiple EDs, such as AN and obesity (Milos et al., 2021; Sader, Waiter, & Williams, 2023), as well as in those experiencing unintentional weight loss (Rönnefarth et al., 2020) or weight gain (Sader, Waiter, & Williams, 2023). Specific contributions concern connectivity of cerebellar regions Crus I/II and Lobule VII (Habas, 2021) with the superior frontal gyrus, coupled with reports that the cerebellum is implicated in goal‐directed behaviour (Li et al., 2021) and emotional regulation (Schmahmann, 2021). These findings provide support that the cerebellum could play a role in ARFID‐aversive symptomatology.

### Study aims

Given the paucity of research in this area, the primary goal of this study is to determine whether altered brain morphology is associated with ARFID symptomatology. Using T_1_‐weighted brain MRI data from the Generation R Study, we assessed brain morphology in children (aged 9–13 years) with versus without ARFID symptoms, classified using DSM‐5‐informed criteria (Sader, Harris, et al., 2023; Sader, Waiter, & Williams, 2023). From existing literature on ARFID and restrictive EDs, we hypothesise that the morphology of the OFC, ACC, cerebellum, insula and superior/middle frontal gyri will be significantly different between those with versus without ARFID symptoms. Specifically, considering prior research of hyperactivation of the insula and OFC in ARFID, as well previous evidence highlighting larger volume of the ACC, OFC and superior/middle frontal gyri in those with AN, we anticipate that these regions will demonstrate larger volume in those with versus without ARFID symptoms. We hypothesise that volume of the cerebellum will be smaller in those with versus without ARFID symptoms due to consistent reports of volumetric reduction in those with various EDs, as well as across BMI ranges.

Due to the aforementioned reported relationship between brain structure and BMI (Steegers et al., 2021), it is also possible that any observable alterations in brain volume may be explained by differences in BMI or states of malnutrition as opposed to ARFID symptomatology alone. Differences in brain morphology have been associated with states of malnutrition in restrictive EDs (Milos et al., 2021), however those with ARFID reportedly include individuals across the BMI‐spectrum (APA, 2013), and as such associations between BMI and brain morphology may not as strongly underly the FED. As such, we also repeated analyses controlling for BMI, hypothesising that the a priori regions will remain significant following BMI correction, supporting specificity of ARFID‐related brain regions.

## Methodology

### Study population

**Figure 1:**
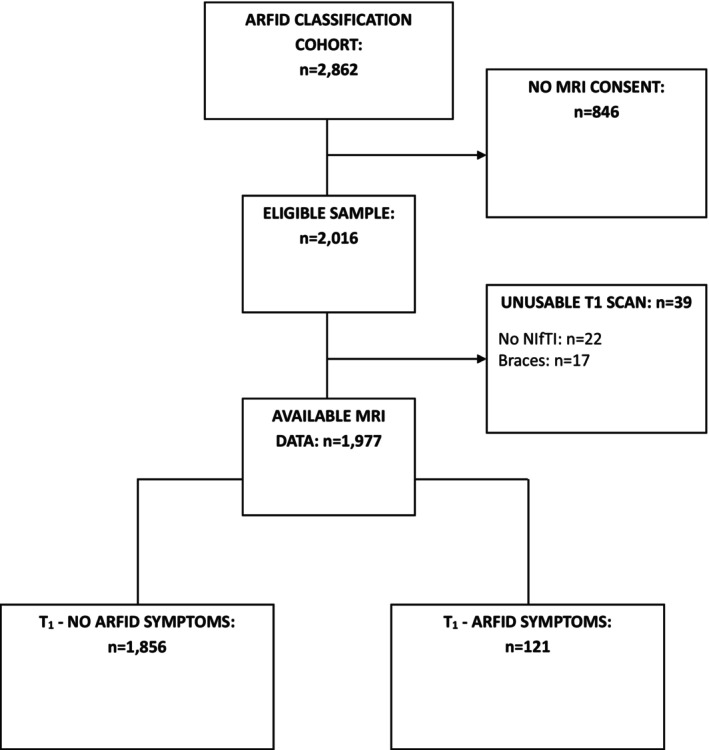
Flow diagram of study participants with available imaging data from previously available ARFID Index classifications (Sader, Harris, et al., 2023; Sader, Waiter, & Williams, 2023). ARFID, avoidant restrictive food intake disorder; MRI, magnetic resonance imaging; NIfTI, neuroimaging informatics technology initiative

The study sample was derived from the Generation R Study (Jaddoe et al., 2010), a population‐based cohort from early pregnancy onwards in Rotterdam, the Netherlands. In total, 9,901 mothers and pregnant women with a delivery date between April 2002 and January 2006 enrolled. Informed, written consent was provided by all participants. The Generation R Protocol was approved by the Medical Ethical Committee of the Erasmus Medical Centre, Rotterdam. We included parents who provided full consent for use of their child's data at age 10 (*n* = 6,036). The Generation R Study is a large, population‐based study of child development with data collection occurring within specific age ranges. Data collection at 10 years was the only wave containing both measures of ARFID and available MRI scans, which were collected on a single, research dedicated MRI system (White et al., 2018). Of these, *n* = 2,862 children were classified as presenting with versus without ARFID symptoms via the ARFID Index (Sader, Harris, et al., 2023; Sader, Waiter, & Williams, 2023). Available data were further filtered for MRI consent (excluded *n* = 846), Neuroimaging Informatics Technology Initiative (NIfTI) file availability (excluded *n* = 22), and presence of braces (excluded *n* = 17). Children with missing data were excluded from further analysis (Figure 1).

### ARFID assessment

Determination as to whether children were classified with ARFID symptomatology was carried out using the method described by Sader, Harris, et al. (2023), Sader, Waiter, and Williams (2023). In brief, children were categorised based on 5 criteria reflecting existing DSM‐5 ARFID symptoms: (1) Avoidant Restrictive Food Intake; (2) Failure to Achieve Expected Weight Gain/Growth; (3) Nutritional Deficiency; (4) Interference with Psychosocial Functioning; and (5) Absence of Body Shape or Weight Concern (Table S1). Children were categorised as presenting with ARFID symptoms if they met Criterion 1 and 5, as well as one (or more) of Criterion 2, Criterion 3 and Criterion 4. Children who did not fulfil these ARFID‐like criteria were categorised as children without ARFID symptoms.

### Sociodemographic information

Sociodemographic information of the parents and participants of this study was collected through previous data collection waves (Jaddoe et al., 2010; White et al., 2018). Weight and height were measured at a research visit at age 9 years. Child age‐ and sex‐specific BMI standard deviation scores (BMI‐SDS) and height‐for‐age SDS were generated using a Dutch reference population (Fredriks et al., 2000).

Covariates for this study included age at assessment based on the date of birth, sex assigned at birth, country of origin, maternal education, and household income. Country of origin, education level, and mean annual household income of parents were collected by questionnaires and categorised following Statistics Netherlands (CBS, 2004; Centre for Research and Statistics, Rotterdam (COS), 2005; Troe et al., 2007). Child country of origin was derived from the parents' country of birth, partitioned into Dutch (including Dutch Antilles), Other Western (American Western, Asian Western, European) and Non‐Western (African, American Non‐Western, Asian Non‐Western, Cape Verdean, Indonesian, Moroccan, Oceania, Surinamese and Turkish). Maternal education was subdivided into Low (up to a high school degree) and High (college/university attendance and higher). Household income was divided into Low (≤2,400 euro per month) and High (>2,400 euro per month).

### Imaging parameters

MRI scans were acquired when the children were ~10 years‐of‐age on a 3.0 Tesla GE Discovery MR750w MRI system operating an eight‐channel head coil (General Electric Healthcare, Milwaukee, WI). Structural imaging parameters were collected via 3D acquisition using an inversion recovery‐prepared fast spoiled gradient recalled sequence (White et al., 2013, 2018).

T_1_‐structural MR images were processed at the time of collection by the Generation R Study using the FreeSurfer Software, version 6.0 (http://surfer.nmr.mgh.harvard.edu). Pre‐processing steps include removal of non‐brain tissue, Talairach transformation, segmentation of grey matter volume (GMV), white matter volume (WMV) and cerebrospinal fluid, differentiation of the cortical GMV/WMV boundary, and grey/pial surface boundary through tessellation and surface deformation. The FreeSurfer image processing steps resulted in multiple volumetric and surface‐based brain global and local brain metrics (i.e. volume, surface area, cortical thickness). Further details of technical procedures are described in previous studies (Muetzel et al., 2019; Steegers et al., 2021).

### Statistical analysis

Analyses were conducted using the R Software (https://cran.r‐project.org/mirrors.html) and SPSS (SPSS Inc. Released 2007. SPSS for Windows, Version 16.0. Chicago, IL). To establish a neuroanatomical profile associated with ARFID (aim 1), FreeSurfer values of T_1_‐weighted structural images were allocated according to ARFID Index classifications (Sader, Harris, et al., 2023; Sader, Waiter, & Williams, 2023). Brain volume and surface area for all cortical/subcortical regions outside of global brain volumes (i.e., total GMV, total WMV, frontal volume, etc.) were corrected for individual head size by establishing proportional ratios of [*x*] region by total estimated intracranial volume (eTIV). The mean volume values between children with versus without ARFID symptoms were compared using Student's *t*‐tests for differences in means via a linear model approach, with effect sizes reported via Cohen's *D* and thresholds of *p* < .05. Linear regression analyses were used to measure the relationship between the defined dependent and independent variables (with brain parameter variables specified for each analysis). Covariates in all analyses included child age, sex, country of origin, maternal education, and household income. A priori analyses focused on hypothesised brain regions implicated in the presentation of ARFID symptoms and appetite restriction across literature, honing in on the ACC, OFC, superior/middle frontal gyri, insula and cerebellum, and additional exploratory analyses consisted of all brain regions. For both a priori and exploratory analyses, correction for multiple comparisons was performed using the false discovery rate (FDR; Benjamini & Hochberg, 1995).

Outside of disorder‐specific symptomatology, the relationships between malnourished states and brain volume have been found in restrictive EDs such as AN (Dreier et al., 2021). To assess whether any existing effects are not predominantly driven by BMI, tests were replicated and controlled for age‐ and sex‐adjusted BMI‐SDS. All analyses were also adjusted for a priori selected covariates (child age, sex, country of origin, maternal education, and household income). Missing data on ARFID Index criteria were imputed using the ‘mice’ package using alternative indicator variables, resulting in *n* = 50 imputed datasets, with missing data on covariates of interest ranging from 0.0% to 9.3% (see Sader, Harris, et al., 2023; Sader, Waiter, & Williams, 2023).

## Results

### Demographics

The final sample size for this study included 1,977 children (ARFID Symptoms = 121/6.1%; no‐ARFID Symptoms = 1,856/93.9%). There were statistically significant differences between children with versus without ARFID symptoms, with the ARFID symptom group having lower birth weight (*p*[FDR] = .0302; *d* = −0.22), age‐ and sex‐adjusted BMI‐SDS (*p*[FDR] = 6.12E‐11; *d* = −0.64), diet quality (*p*[FDR] = 2.40E‐14; *d* = −0.74) and energy intake (in kcal; *p*[FDR] = 5.86E‐04; *d* = −0.34). In addition, the ARFID symptom group had greater picky eating scores (*p*[FDR] = 1.80E‐15; *d* = 1.87) and psychosocial impact of ARFID symptomatology (on Family Meals [*p*(FDR) = 1.80E‐15; *d* = 1.65], Learning/Working [*p*(FDR) = 9.00E‐13; *d* = 0.33], Playing/Hobbies [*p*(FDR) = 5.64E‐04; *d* = 0.35]). Comparing children with versus without ARFID symptoms, there were no observable differences between sex, country of origin, household income, and maternal education/BMI (Table 1).

**Table 1:** Demographic characteristics between those with (*n* = 121) and those without (*n* = 1,856) ARFID symptoms

Characteristics	Total	ARFID S.	No ARFID S.	*t*/*p* ^a^	*p*(FDR)	*d*
Sex (*N*; % boys)	1,977; 49.67%	121; 51.24%	1,856; 49.57%	*p* = .7931	8.40E‐01	0.03
Country of origin^a^ (*N*; %)						
Dutch	1,455; 73.60%	88; 72.73%	1,367; 73.65%	*p* = .9065	9.07E‐01	−0.07
Other Western	175; 8.85%	7; 5.79%	168; 9.05%	*p* = .2889	3.47E‐01	
Non‐Western	344; 17.40%	26; 21.49%	318; 17.13%	*p* = .2712	3.47E‐01	
Birth weight (g)	**3,450.04 ± 561.23**	**3,334.83 ± 602.73**	**3,457.50 ± 557.79**	***t* = −2.431**	3.02E‐02*	**−0.22**
BMI‐SDS, 10y	**0.12 ± 0.95**	**−0.44 ± 1.09**	**0.16 ± 0.93**	***t* = −6.77**	6.12E‐11***	**−0.64**
Mean Picky Eating Sum Score (SFQ); SR = 0–20	**9.14 ± 3.43**	**14.64 ± 2.10**	**8.78 ± 3.19**	***t* = 19.94**	1.80E‐15***	**1.87**
Mean Diet Quality Score (FFQ); SR = 0–10	**4.57 ± 1.19**	**3.76 ± 1.22**	**4.62 ± 1.17**	***t* = −7.917**	2.40E‐14***	**−0.74**
Mean Energy Intake (kcal)	**1,477.46 ± 349.78**	**1,367.72 ± 340.92**	**1,484.61 ± 349.25**	***t* = −3.693**	5.86E‐04**	**−0.34**
Mean Psychosocial Impact Item (DAWBA); SR = 0–15						
Family meals	**1.36 ± 0.74**	**2.42 ± 1.08**	**1.29 ± 0.65**	***t* = 17.59**	1.80E‐15***	**1.65**
Learning/Working	**1.14 ± 0.47**	**1.28 ± 0.66**	**1.13 ± 0.45**	***t* = 3.548**	9.00E‐13***	**0.33**
Playing/Hobbies	**1.22 ± 0.61**	**1.42 ± 0.81**	**1.21 ± 0.59**	***t* = 3.741**	5.64E‐04**	**0.35**
Mean bodyweight concern (DAWBA); SR = 0–2	**1.30 ± 0.47**	**1.00 ± 0.00**	**1.32 ± 0.48**	***t* = −7.39**	9.36E‐13***	**−0.69**
Parental characteristics						
Maternal education^b^ (*N*; %)						
High	1,351; 68.34%	76; 62.81%	1,275; 68.70%	*p* = .2121	3.20E‐01	
Low	577; 29.19%	45; 37.19%	532; 28.66%	*p* = .058	1.04E‐01	
Household income						
<2,400 EUR	323; 16.34%	25; 20.66%	298; 16.06%	*p* = .2299	3.20E‐01	−0.13
>2,400 EUR	1,533; 77.54%	88; 72.73%	1,445; 77.86%	*p* = .2312	3.20E‐01	
BMI mother at recruitment (weight/height^2^)	24.08 ± 3.75	23.95 ± 3.17	24.08 ± 3.79	*t* = −0.32	8.39E‐01	−0.03

Regions in bold indicate a significant difference between those with versus those without ARFID symptoms, as indicated by *T* values used for Student's *t*‐test, and *p*‐values used for two‐proportion *z* tests. ARFID, avoidant restrictive food intake disorder; *d*, Cohen's *D* effect size; DAWBA, Development and Well‐Being Assessment; EUR, euro; FDR, false discovery rate; S., symptoms; SR, score range.

^a^ Country of origin is defined as follows: Dutch (including Dutch Antilles); Other Western (American Western, Asian Western, European); Non‐Western (African, American Non‐Western, Asian Non‐Western, Cape Verdean, Indonesian, Moroccan, Oceania, Surinamese and Turkish).

^b^ Maternal education is defined as follows: Low (up to a high school degree); High (college/university attendance and higher).

*p(FDR) < 0.05; **p(FDR) < 0.01; ***p(FDR) < 0.001.

#### Brain morphology comparisons: Hypothesis‐driven analysis

**Figure 2:**
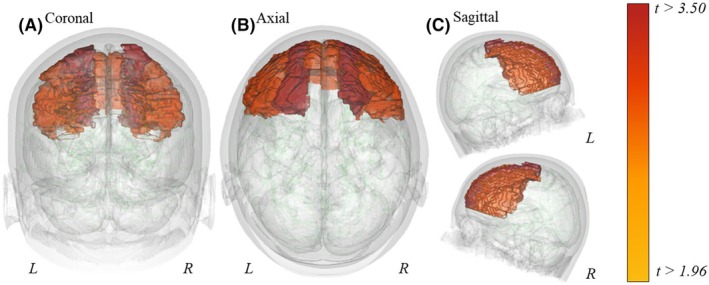
Hypothesised and exploratory brain regions exhibiting greater cortical thickness in those with (*n* = 121) versus without (*n* = 1,856) ARFID symptoms. Subfigures A, B and C respectively display coronal, axial and sagittal orientations. Those with ARFID symptoms exhibited greater mean thickness in the bilateral superior frontal (red) and frontal (dark orange) cortices. L, left; R, right

Evaluating the volume, mean thickness, and surface area of hypothesised brain regions identified greater thickness in the frontal (*p* = .00743; *p*[FDR] = 0.0334; *d* = 0.21) and superior frontal lobes (*p* = 6.56E‐04; *p*[FDR] = .00590; *d* = 0.28) in those with ARFID symptoms (Figure 2; Table 2). No observable differences in the regional surface area or volume of hypothesised regions were identified.

**Table 2:** A priori cortical thickness, surface area, and volume of those with (*n* = 121) versus without (*n* = 1,856) ARFID symptoms

	*M* ± *SD* ARFID S. (mm^3^)	*M* ± *SD* No ARFID S. (mm^3^)	*t* (Tukey)^a^	*p*	*d* ^a^
Region – thickness					
MF Caudal	2.74 ± 0.14	2.72 ± 0.13	−1.849	0.0647	−0.13
MF Rostral	2.66 ± 0.12	2.64 ± 0.11	−2.256	0.0242	−0.19
**Superior frontal**	**3.02 ± 0.13**	**3.00 ± 0.12**	**−3.413**	0.000656**	**−0.28**
**Frontal**	**2.80 ± 0.10**	**2.78 ± 0.09**	**−2.68**	0.00743*	**−0.21**
ACC Caudal	2.87 ± 0.20	2.88 ± 0.20	0.455	0.649	0.05
ACC Rostral	3.30 ± 0.17	3.30 ± 0.18	−0.264	0.792	−0.01
Cingulate	2.89 ± 0.11	2.89 ± 0.11	−0.254	0.8	0.03
OFC Lateral	2.93 ± 0.12	2.92 ± 0.13	−0.962	0.336	−0.11
OFC Medial	2.78 ± 0.14	2.75 ± 0.15	−2.108	0.0352	−0.17
Insula	3.25 ± 0.14	3.24 ± 0.13	−0.190	0.850	−0.02
Region – Surface area^**b**^					
MF Caudal	0.00158 ± 0.000197	0.00158 ± 0.000193	0.505	0.614	0.015
MF Rostral	0.00417 ± 0.000476	0.000415 ± 0.000447	−0.129	0.897	−0.062
Superior Frontal	0.000513 ± 0.000448	0.00514 ± 0.000433	0.622	0.534	0.04
Frontal^b^	31956.76 ± 3538.78	32178.46 ± 3601.77	0.878	0.38	0.06
ACC caudal	0.000447 ± 6.31E‐05	0.000450 ± 6.29E‐05	0.713	0.476	0.05
ACC rostral	0.000488 ± 7.52E‐05	0.000495 ± 7.20E‐05	0.986	0.324	0.1
Cingulate	0.00249 ± 0.000211	0.00248 ± 0.000203	0.389	0.697	−0.02
OFC lateral	0.00181 ± 0.000198	0.00182 ± 0.000200	0.831	0.406	0.07
OFC medial	0.00130 ± 0.000132	0.00130 ± 0.000129	0.482	0.63	0.02
Insula	0.00157 ± 0.000142	0.00158 ± 0.000133	−0.195	0.845	0.09
Region – Volume					
CC posterior	0.000562 ± 7.66E‐05	0.000559 ± 8.54E‐05	−0.251	0.802	−0.03
CC Mid‐posterior	0.000324 ± 4.98E‐05	0.000325 ± 5.69E‐05	0.023	0.982	0.02
CC central	0.000365 ± 9.20E‐05	0.000363 ± 8.77E‐05	0.117	0.907	−0.02
CC Mid‐anterior	0.000377 ± 1.012E‐04	0.000369 ± 9.56E‐05	−0.735	0.462	−0.09
CC anterior	0.000574 ± 8.74E‐05	0.000568 ± 8.52E‐05	−0.849	0.396	−0.07
CB GM	0.0392 ± 0.00304	0.0392 ± 0.00313	0.089	0.929	0
CB WM	0.00850 ± 0.000768	0.00857 ± 0.000849	0.673	0.501	0.09
Insula	0.00534 ± 0.000453	0.00538 ± 0.000438	−0.146	0.884	0.09

Regions in bold indicate a significant difference between those with versus those without ARFID symptoms. ACC, anterior cingulate cortex; ARFID, avoidant restrictive food intake disorder; CB, cerebellum; CC, cingulate cortex; GM, grey matter; M, mean; MF, middle frontal; OFC, orbitofrontal cortex; SD, standard deviation; WM, white matter.

^a^ *T* and *d* values reported as No ARFID S. – ARFID S.

^b^ Marked regions are not corrected for head size.

*p(FDR) < 0.05, **p(FDR) < 0.01.

#### Brain morphology comparisons: Exploratory analysis and BMI adjustment

Exploratory analysis of thickness, surface area, and volume identified regions with aberrant structure in those with ARFID symptoms relative to those without, but none survived the FDR correction (Table S2). Additional correction for BMI identified very slight changes in a priori thickness in those with versus those without ARFID symptoms (Table S3). Significance as reported in both *p* and Cohen's *d* values was marginally greater within the frontal cortex (*p*[FDR] = .0334/*d* = 0.21 without BMI correction to *p*[FDR] = .0277/*d* = 0.21) and marginally smaller within the superior frontal cortex (*p*[FDR] = .00590/*d* = 0.28 to *p*[FDR] = .00800/*d* = 0.28).

## Discussion

This large‐scale structural MRI study evaluated and compared the brain morphology of 121 children with and 1,856 children without ARFID symptoms. Those with ARFID symptoms presented with significantly greater superior frontal/frontal cortical thickness, which were unaffected upon adjustment for BMI. Effect sizes reported for these regions are relatively small (0.21–0.28). While subsequent clinical implications are unclear, significance of these findings may be further pronounced in the study samples evaluating clinically diagnosed ARFID populations and serve as a basis for future research. The extent and direction of these findings was anticipated due to (1) previous ARFID literature implicating the PFC (Thomas et al., 2017); (2) studies in restrictive EDs reporting increased volume of superior/middle frontal gyri (Sader et al., 2022); (3) neuroimaging research in autistic cohorts that exhibit elevated co‐occurrence with ARFID (Sanchez‐Cerezo, Nagularaj, Gledhill, & Nicholls, 2023), identifying increased thickness of the frontal cortex (Zielinski et al., 2014). Aside from these regions, other a priori hypothesised regions, such as the insula, ACC, OFC, and cerebellum were not found to significantly differ between children with versus without ARFID symptomatology and suggest that these regions may not be as prominently implicated in ARFID symptomatology.

### Anatomical comparison: Children with versus without ARFID symptoms

Those with versus without ARFID symptomatology demonstrated significantly larger regional mean thickness volume, including the superior frontal/frontal cortices. The superior frontal cortices are implicated in cognitive and executive functions including working memory (Kragel et al., 2018) and perceptual reasoning/impulsiveness (Schilling et al., 2013). Medial frontal regions have been linked with action/performance (Ridderinkhof, Ullsperger, Crone, & Nieuwenhuis, 2004) and action (Luu, Flaisch, & Tucker, 2000) monitoring, as well as with representations of negative emotions (Kragel et al., 2018). Greater relative volumes of frontal regions were recently reported in other FEDs such as AN (Leppanen, Sedgewick, Cardi, Treasure, & Tchanturia, 2019; Sader et al., 2022), and it is possible that such alterations in executive and emotional regions seen in those with ARFID may be associated with appetitive and behavioural symptoms characteristic of the disorder.

Findings on the drivers and consequences of altered cortical thickness obtained from the literature provides a tentative theory of how thickness of the cortex is associated with ARFID symptoms in children. The cerebral cortex contains a dense arrangement of cells divided into six subsections, which function to receive inputs and communicate with distant brain regions (Sowell et al., 2004). In typical populations, greater thickness of cortical grey matter, particularly prefrontal cortical thickness, undergoes rapid development in children aged 5–11 years (Sowell et al., 2004). Given our findings of larger frontal mean cortical thickness in those with ARFID symptoms, it is possible that disorder‐specific symptomatology may be associated with altered thickness across cortical layers, or altered communication with different brain regions, such as the OFC which plays significant roles in valence‐based decision‐making via connections with the medial prefrontal cortex (Rolls et al., 2020).

Thickening of the frontal cortex in ARFID may also indicate a developmental disruption, as seen in other psychological disorders. During typical neurodevelopment, synaptic pruning and apoptosis (or programmed cell death) occur, in which synapses and/or entire neurons respectively are eliminated through microglia‐mediated processes (Paolicelli et al., 2011). Dysregulation of synaptic pruning has been implicated across a variety of neuropsychiatric disorders such as schizophrenia (Osimo, Beck, Reis Marques, & Howes, 2019) and bipolar disorder (Zoghbi & Bear, 2012), or neurodevelopmental disorders such as autism (Waiter et al., 2004; Zoghbi & Bear, 2012) and attention deficit hyperactivity disorder (Zoghbi & Bear, 2012). Greater thickness and proposed apoptotic/synaptic pruning impairments have been reported in studies with autistic individuals (Zielinski et al., 2014). This fits the observation that autistic individuals also display high rates of comorbidity with ARFID, with prevalence rates ranging between 21% and 28% (Sanchez‐Cerezo et al., 2023). Increases in spine density have been associated with autistic behaviours as well as hyperconnectivity of the cortico‐striatal pathway (Pagani et al., 2021), which serves important roles in the development of motivational and cognition‐related goal‐oriented behaviours. Brain regions central to this pathway include the dorsal prefrontal cortex (PFC), dorsal ACC, prefrontal cortex and OFC (Haber, 2016), of which we found the PFC to be larger in those with ARFID symptoms. Thus, volumetric alterations seen in the presence of ARFID symptomatology may suggest discrepant neuroanatomical structure across development compared with typical populations.

Greater thickness of the frontal lobes may also suggest that mechanisms associated with ARFID are related to regions implicated in conflict anticipation, orientation of attentional resources and inhibition control. Including roles associated with anticipation and monitoring of conflict (Berkman et al., 2012), the superior frontal lobes are well known for their roles in inhibitory function (Floden & Stuss, 2006; Gavazzi, Giovannelli, Currò, Mascalchi, & Viggiano, 2021). Mechanisms contributing to inhibition have been classified as ‘proactive’ and 'reactive’ (Gavazzi et al., 2021). Proactive inhibition involves top‐down mechanisms of control, working to plan actions and suppress pre‐emptive actions prior to the occurrence of specific actions (Gavazzi et al., 2021). Reactive inhibition, an alternate response, encompasses a bottom‐up measure of control, and is suggested to function as a trigger to stop already initiated motor functions (Gavazzi et al., 2021). Aside from roles in processing attention (Japee et al., 2015) and context‐dependent goal representations (Turnbull et al., 2019), the middle frontal gyrus has also been shown to be associated with both reactive and proactive means of inhibitory control (Gavazzi et al., 2021). Additionally, cortical thickness within the superior (Schilling et al., 2013) and middle (Pan et al., 2021) frontal regions has inversely correlated with traits of impulsivity, and are suggested to act as ‘brakes’ towards impulsive tendencies via the exertion of inhibitory control (Pan et al., 2021). Future task‐based functional imaging studies in ARFID are warranted to test this hypothesis.

Importantly, the current cross‐sectional study design is unable to infer causal brain‐behaviour relationships. It is unclear whether the neuroanatomical differences between children with versus without ARFID symptoms are a cause, consequence, or correlate of ARFID symptomatology. For instance, it is unknown as to whether nutritional deficiencies or other associated ARFID symptoms precede, or are subsequent to, neuroanatomical changes.

### Strengths and limitations

This study has several strengths, such as utility of a large, population‐based sample of children, which increases the generalisability of the findings. We also had a vast array of sociodemographic, behavioural, appetitive, anthropometric and structural imaging data to account for potential biases. Variables, particularly covariates used for analysis consisted of sociodemographic factors that are highly reproducible and can be incorporated into future research. Lastly, this study provided an in‐depth analysis of multiple structural brain parameters and investigates parameters beyond volumetric structure (i.e. cortical thickness, surface area).

There are also a number of limitations to our study. First, not all participants were included from our initial classification study (Sader, Harris, et al., 2023; Sader, Waiter, & Williams, 2023) due to not all children receiving an MRI, unusable MRIs, alternative imaging parameters (*n* = 22), and the presence of individuals with braces (*n* = 17). Despite reductions in sample size, population‐based characteristics of our sample parallel those seen during initial classification. As the study population consisted of those aged 10 years, findings are not representative of older individuals with ARFID symptoms. The MRI assessment also occurred at a different timepoint than collection of some ARFID Index evaluation measures, such as collection of diet quality scores obtained at age 8 for Criterion 3 of the Index. However, we attempted to control for this by adjusting for child age across analyses. Similarly, as brain maturation occurs quickly throughout childhood, we cannot generalise findings to younger children. This study utilised a cross‐sectional design, and thus, we are unable to determine the directionality or causality of brain‐behaviour effects. Further functional and longitudinal‐based work would greatly assist in validating the directionality of effects across neuroanatomy and ARFID‐related behaviour.

Due to a lack of (semi)‐structured DSM‐5 diagnostic interviews focused specifically on ARFID within the Generation R Study, the current study applied ARFID Index categorisations to measure ARFID symptomatology based on DSM‐5‐based criteria (Sader, Harris, et al., 2023, Sader, Waiter, & Williams, 2023). As such, the work in this study is unable to provide conclusive information regarding neural correlates associated with a clinical ARFID diagnosis, meaning the full generalisability to youth formally diagnosed with ARFID is unknown. Additionally, the work does not account for existing ARFID profiles (ARFID‐limited intake, ARFID‐limited variety, and ARFID‐aversive; APA, 2022). Lastly, future research could consider developing a continuous measurement of ARFID severity at the population level. Despite limitations, this study offers a widespread view and understanding of novel neuroanatomical findings present within ARFID using large‐scale population‐based means.

## Conclusion

To the best of our knowledge, this is the first large‐scale structural MRI study of ARFID to date, utilising 1,977 children aged 10 years from the Dutch Generation R Study. Those with (*n* = 121) versus without (*n* = 1,856) ARFID symptoms exhibited greater frontal cortical thickness. Findings suggest: (1) Cortical thickness of the frontal/superior frontal lobes may represent neural correlates specific to ARFID symptomatology; (2) Children with ARFID symptoms may experience neurodevelopmental differences not present within typical populations, which could serve to reinforce avoidant/restrictive feeding behaviours. Anatomical findings in this study act as foundational groundwork for future functional and structural imaging research into ARFID cohorts, and suggest the implementation of clinical treatment outside of appetitive domains for those with ARFID.

## Ethical considerations

The Generation R Study team worked to ensure sex and gender balance in the recruitment of human participants, as well as to ensure race, ethnic, and/or other types of diversity in the recruitment of human participants. While citing references scientifically relevant for this work, the author team associated with this article also actively worked to promote inclusion of historically underrepresented racial and/or ethnic groups in science in our reference list. The author list of this article includes contributors from the location and/or community where the research was conducted who participated in the data collection, design, analysis, and/or interpretation of the work. The research team also actively worked to promote sex and gender balance in the author group.

Written informed consent was provided by all participants. The Generation R Protocol was approved by the Medical Ethical Committee of the Erasmus Medical Centre, Rotterdam.

**Key points**

- Prior to this study, no research on the structural neuroanatomy associated with avoidant restrictive food intake disorder (ARFID) has been conducted.
- This large‐scale study included 1,977 children aged 10 years who were evaluated according to the presentation of ARFID symptomatology using the ARFID Index.
- We identified greater superior frontal/frontal cortex thickness in children with versus without ARFID symptomatology.
- Findings suggest ARFID‐specific symptomatology is associated with altered regional cortical thickness, irrespective of BMI.
- Implicated neural correlates are associated with neurocircuitry important for executive function, suggesting that impairments of development may be important in the aetiology of this condition.

## Supporting information


**Table S1**. ARFID Index characteristics and cut‐off scores, including subject age and reporter information from evaluation tools.
**Table S2**. Exploratory cortical thickness, surface area and volume of those with (*n* = 121) versus without (*n* = 1,856) ARFID symptoms.
**Table S3**. Exploratory cortical thickness, surface area and volume of those with (*n* = 121) versus without (*n* = 1,856) ARFID symptoms post‐BMI correction.

## Data Availability

The data that support the findings of this study are available upon reasonable request to the data management team and management team of the Generation R Study (datamanagementgenr@erasmusmc.nl).
