# Supplementary material for: Neural correlates of children with avoidant restrictive food intake disorder symptoms: large‐scale neuroanatomical analysis of a paediatric population
Source: J Child Psychol Psychiatry. 2024 Dec 2;66(6):785–95. doi: 10.1111/jcpp.14086 (PMC12062856; doi:10.1111/jcpp.14086)
Supplement: Supplementary file 1 — Table S1. ARFID Index characteristics and cut‐off scores, including subject age and reporter information from evaluation tools. Table S2. Exploratory cortical thickness, surface area and volume of those with (n = 121) versus without (n = 1,856) ARFID symptoms. Table S3. Exploratory cortical thickness, surface area and volume of those with (n = 121) versus without (n = 1,856) ARFID symptoms post‐BMI correction. [file JCPP-66-785-s001.docx]

***Neural Correlates of Children with Avoidant Restrictive Food Intake Disorder (ARFID) Symptoms: Large-Scale Neuroanatomical Analysis of a Paediatric Population***

**SUPPLEMENTARY MATERIAL**

*2.2 – ARFID Assessment*

| **Table S1. ARFID Index characteristics and cut-off scores, including subject age and reporter information from evaluation tools**  *****A child classifies as presenting with ARFID symptoms if s/he meets Criterion 1 and 5, *and* at least one sub-criteria (2-4) | | | | | |
| --- | --- | --- | --- | --- | --- |
| **Index Criterion** | **Child Age (Years)** | | **Reporter** | **Evaluation Tool** | **Classification for Meeting Criteria** |
| *1. Avoidant/Restrictive Food Intake* | | 10 | Mother | **SFQ**; 4-item picky eating scale | ≥12/20 (Corresponds to ≥ 'Sometimes' for all items) |
| *2. Failure to Achieve Expected Weight Gain/Growth* | | 10 | Researcher - Measured | BMI categorised following **IOTF** classifications | ≤TG1 (≤-1 SD) |
| *3. Nutritional Deficiency* | | 8 | Mother | **FFQ**: - Diet Quality score AND - Energy Intake (kcal/d) | ≤20th percentile for Diet Quality AND Energy Intake |
| *4. Interference with Psychosocial Functioning* | | 10 | Mother | **3 DAWBA items**: "*How often does your child's eating behaviour disrupt (****4a****) family meals, (****4b****) learning or working at school and (****4c****) playing, hobbies, sports or other leisure activities?*" (5-point scale) | ≥"Sometimes" on at least 1 item (≥3/15) |
| *5. Absence of Body Shape/Weight Dissatisfaction* | | 10 | Mother | **1 DAWBA item**: "*Is your child worried about gaining weight or becoming fat?*" (3-point scale) | "No" vs. "Yes"/"A Little Bit" (≤1/3) |

*Footnotes:* [*Abbreviations: ARFID – Avoidant/Restrictive Food Intake Disorder; BMI – Body Mass Index; DAWBA – Development and Wellbeing Assessment; FFQ – Food Frequency Questionnaire; IOTF – International Obesity Task Force; SFQ – Stanford Feeding Questionnaire; TG – Thinness Grade*].

*2.5 – Statistical Analysis*

Brain images were generated and constructed using the “misc3d” and “neurobase” packages on the R Software (Version 4.2.1.).

*3.2.2. – Brain Morphology Comparisons: Exploratory Analysis*

| **Table S2. Exploratory cortical thickness, surface area and volume of those with (n=121) vs. without (n=1,856) ARFID symptoms** | | | | | |
| --- | --- | --- | --- | --- | --- |
| **Region - Thickness** | **M±SD ARFID S. (mm^3^)** | **M±SD NO ARFID S. (mm^3^)** | **t (Tukey)^a^** | **p** | ***d*^a^** |
| MF Caudal | 2.74±0.14 | 2.72±0.13 | -1.849 | 0.0647 | -0.13 |
| **MF Rostral** | **2.66±0.12** | **2.64±0.11** | **-2.256** | **0.0242** | -0.19 |
| Banks STS | 2.83±0.15 | 2.81±0.15 | -1.92 | 0.0551 | -0.15 |
| Entorhinal | 3.25±0.32 | 3.25±0.31 | 0.347 | 0.729 | 0.02 |
| Fusiform | 2.93±0.11 | 2.92±0.11 | -0.754 | 0.451 | -0.06 |
| Inferior Parietal | 2.74±0.12 | 2.74±0.12 | -1.523 | 0.128 | -0.08 |
| Inferior Temporal | 2.78±0.15 | 2.77±0.13 | -1.22 | 0.223 | -0.09 |
| Lateral Occipital | 2.25±0.10 | 2.25±0.11 | -0.035 | 0.972 | 0 |
| Lingual | 2.18±0.13 | 2.17±0.13 | -1.014 | 0.311 | -0.05 |
| **Middle Temporal** | **2.98±0.17** | **2.95±0.14** | **-2.235** | **0.0255** | -0.19 |
| Parahippocampal | 3.00±0.20 | 3.01±0.22 | 0.281 | 0.779 | 0.07 |
| Paracentral | 2.71±0.12 | 2.70±0.12 | -1.244 | 0.214 | -0.09 |
| Precentral | 2.62±0.11 | 2.61±0.11 | -1.193 | 0.233 | -0.07 |
| Postcentral | 2.20±0.11 | 2.22±0.11 | 0.581 | 0.561 | 0.1 |
| Precuneus | 2.79±0.09 | 2.78±0.10 | -1.828 | 0.0677 | -0.12 |
| Cuneus | 2.08±0.14 | 2.08±0.15 | -0.301 | 0.763 | 0 |
| **Superior Frontal** | **3.02±0.13** | **3.00±0.12** | **-3.413** | **0.000656*** | **-0.28** |
| Superior Parietal | 2.42±0.11 | 2.42±0.11 | -0.781 | 0.435 | -0.01 |
| Superior Temporal | 2.94±0.15 | 2.92±0.14 | -1.721 | 0.0855 | -0.13 |
| **Frontal** | **2.80±0.10** | **2.78±0.09** | **-2.68** | **0.00743** | -0.21 |
| Temporal | 2.92±0.12 | 2.91±0.11 | -1.602 | 0.109 | -0.13 |
| Parietal | 2.58±0.09 | 2.58±0.09 | -1.204 | 0.229 | -0.04 |
| Occipital | 2.14±0.10 | 2.14±0.10 | -0.709 | 0.479 | -0.04 |
| Parsopercularis | 2.84±0.13 | 2.83±0.12 | -1.309 | 0.191 | -0.07 |
| Parsorbitalis | 2.93±0.17 | 2.91±0.17 | -1.768 | 0.0773 | -0.15 |
| Parstriangularis | 2.71±0.14 | 2.71±0.12 | -0.998 | 0.319 | -0.07 |
| Pericalcarine | 1.68±0.15 | 1.67±0.16 | -0.871 | 0.384 | -0.05 |
| Supramarginal | 2.80±0.11 | 2.80±0.11 | -1.421 | 0.156 | -0.07 |
| ACC Caudal | 2.87±0.20 | 2.88±0.20 | 0.455 | 0.649 | 0.05 |
| ACC Rostral | 3.30±0.17 | 3.30±0.18 | -0.264 | 0.792 | -0.01 |
| Cingulate | 2.89±0.11 | 2.89±0.11 | -0.254 | 0.8 | 0.03 |
| Insula | 3.25±0.14 | 3.24±0.13 | -0.203 | 0.839 | -0.02 |
| OFC Lateral | 2.93±0.12 | 2.92±0.13 | -0.962 | 0.336 | -0.11 |
| **OFC Medial** | **2.78±0.14** | **2.75±0.15** | **-2.108** | **0.0352** | -0.17 |
| Transverse Temporal | 2.70±0.18 | 2.68±0.18 | -1.447 | 0.148 | -0.13 |
| Isthmus Cingulate | 2.77±0.18 | 2.77±0.16 | -0.854 | 0.393 | 0 |
| Temporal Pole | 3.39±0.40 | 3.40±0.37 | 0.71 | 0.478 | 0 |
| Frontal Pole | 2.85±0.35 | 2.85±0.34 | 0.084 | 0.933 | 0 |
| **^b^ Region – Surface Area** | **M±SD ARFID (mm^3^)** | **M±SD NO ARFID S. (mm^3^)** | **t (Tukey)^b^** | **p** | ***d*^a^** |
| MF Caudal | 0.00158±0.000197 | 0.00158±0.000193 | 0.505 | 0.614 | 0.015 |
| MF Rostral | 0.00417±0.000476 | 0.000415±0.000447 | -0.129 | 0.897 | -0.062 |
| BanksSTS | 0.000673±8.06E-05 | 0.000671±8.03E-05 | 0.38 | 0.704 | -0.028 |
| Entorhinal | 0.000278±4.86E-05 | 0.000286±5.07E-05 | 1.734 | 0.0831 | 0.17 |
| Fusiform | 0.00214±0.000199 | 0.00215±0.000187 | 0.743 | 0.458 | 0.05 |
| Inferior Parietal | 0.00370±0.000419 | 0.00371±0.000385 | 0.4 | 0.689 | 0.01 |
| Inferior Temporal | 0.00234±0.000299 | 0.00234±0.000252 | 0.726 | 0.468 | -0.01 |
| Lateral Occipital | 0.00397±0.000362 | 0.00392±0.000400 | -0.782 | 0.435 | -0.13 |
| Lingual | 0.00225±0.000256 | 0.00221±0.000260 | -0.789 | 0.43 | -0.14 |
| Middle Temporal | 0.00233±0.000265 | 0.00231±0.000217 | 0.397 | 0.692 | -0.08 |
| Parahippocampal | 0.000443±4.53E-05 | 0.000445±4.26E-05 | 1.486 | 0.137 | 0.05 |
| Paracentral | 0.000999±8.56E-05 | 0.00100±9.23E-05 | 0.652 | 0.515 | 0.03 |
| Precentral | 0.00333±0.000241 | 0.00335±0.000255 | 0.963 | 0.336 | 0.08 |
| Postcentral | 0.00289±0.000257 | 0.00292±0.000242 | 1.681 | 0.0929 | 0.12 |
| Precuneus | 0.00286±0.000263 | 0.00285±0.000256 | 0.334 | 0.738 | -0.02 |
| Cuneus | 0.00112±0.000134 | 0.00112±0.000139 | 0.826 | 0.409 | 0.02 |
| Superior Frontal | 0.000513±0.000448 | 0.00514±0.000433 | 0.622 | 0.534 | 0.04 |
| Superior Parietal | 0.00407±0.000409 | 0.00408±0.000404 | 0.514 | 0.607 | 0.01 |
| Superior Temporal | 0.00258±0.000244 | 0.00260±0.000204 | 1.561 | 0.119 | 0.12 |
| ^b^ Frontal | 31956.76±3538.78 | 32178.46±3601.77 | 0.878 | 0.38 | 0.06 |
| ^b^ Temporal | 17130.63±1916.43 | 17284.54±1872.55 | 1.445 | 0.149 | 0.08 |
| ^b^ Parietal | 24579.91±2710.78 | 24853.80±2781.48 | 1.446 | 0.148 | 0.1 |
| ^b^ Occipital | 12631.51±1311.32 | 12565.65±1481.43 | -0.333 | 0.739 | -0.04 |
| Parsopercularis | 0.00105±0.000124 | 0.00104±0.000123 | -0.047 | 0.962 | -0.03 |
| Parsorbitalis | 0.000539±5.73E-05 | 0.000537±5.77E-05 | 0.219 | 0.827 | -0.03 |
| Parstriangularis | 0.00101±0.000116 | 0.00100±0.000123 | 0.067 | 0.947 | -0.02 |
| Pericalcarine | 0.00103±0.000143 | 0.00102±0.000161 | -0.1 | 0.92 | -0.04 |
| **Supramarginal** | **0.00273±0.000317** | **0.00278±0.000309** | **1.976** | **0.0483** | 0.16 |
| ACC Caudal | 0.000447±6.31E-05 | 0.000450±6.29E-05 | 0.713 | 0.476 | 0.05 |
| ACC Rostral | 0.000488±7.52E-05 | 0.000495±7.20E-05 | 0.986 | 0.324 | 0.1 |
| Cingulate | 0.00249±0.000211 | 0.00248±0.000203 | 0.389 | 0.697 | -0.02 |
| Insula | 0.00157±0.000142 | 0.00158±0.000133 | 0.784 | 0.433 | 0.09 |
| OFC Lateral | 0.00181±0.000198 | 0.00182±0.000200 | 0.831 | 0.406 | 0.07 |
| OFC Medial | 0.00130±0.000132 | 0.00130±0.000129 | 0.482 | 0.63 | 0.02 |
| **Transverse Temporal** | **0.000261±3.04E-05** | **0.000268±3.01E-05** | **2.449** | **0.0144** | 0.43 |
| Isthmus Cingulate | 0.000703±6.61E-05 | 0.000691±7.33E-05 | -0.786 | 0.432 | -0.16 |
| Temporal Pole | 0.000298±3.59E-05 | 0.000296±3.33E-05 | -0.135 | 0.893 | -0.07 |
| Frontal Pole | 0.000209±1.99E-05 | 0.000208±2.09E-05 | -0.32 | 0.749 | -0.04 |
| **^b^ Region - Volume** | **M±SD ARFID S. (mm^3^)** | **M±NO ARFID S. (mm^3^)** | **t (Tukey)^a^** | **p** | ***d*^a^** |
| ^b^ eTIV | 1512370.0±125440.8 | 1522073.0±139557.5 | 0.853 | 0.394 | 0.07 |
| ^b^ GM | 289907.9±28027.3 | 290990.2±27982.1 | 0.486 | 0.627 | 0.04 |
| ^b^ GM Cortex | 579815.8±56054.6 | 581980.3±55964.3 | 0.486 | 0.627 | 0.04 |
| ^b^ GM Subcort | 60276.3±4170.5 | 60731.5±4610.4 | 1.02 | 0.308 | 0.1 |
| ^b^ GM Total | 759353.0±65780.1 | 762641.4±66296.2 | 0.633 | 0.527 | 0.05 |
| ^b^ WM | 210866.8±24107.3 | 211967.7±25362.2 | 0.66 | 0.51 | 0.04 |
| ^b^ WM Total | 421733.5±48214.6 | 423935.4±50724.4 | 0.66 | 0.51 | 0.04 |
| Brainstem | 0.0123±0.000785 | 0.0123±0.000939 | -0.126 | 0.9 | -0.01 |
| CSF | 0.000600±0.000129 | 0.000600±0.000120 | 0.244 | 0.807 | 0 |
| Ventral DC | 0.00269±0.000191 | 0.00269±0.000190 | 0.476 | 0.634 | 0.05 |
| Vessel | 2.02E-05±8.52E-06 | 2.11E-05±1.15E-05 | 0.421 | 0.674 | 0.08 |
| Choroid Plexus | 0.000274±6.71E-05 | 0.000274±6.66E-05 | -0.127 | 0.899 | 0 |
| ^b^ **Mean Thickness** | **2.69±0.081** | **2.68±0.079** | **-1.973** | **0.0486** | -0.13 |
| CC Posterior | 0.000562±7.66E-05 | 0.000559±8.54E-05 | -0.251 | 0.802 | -0.03 |
| CC Mid Posterior | 0.000324±4.98E-05 | 0.000325±5.69E-05 | 0.023 | 0.982 | 0.02 |
| CC Central | 0.000365±9.20E-05 | 0.000363±8.77E-05 | 0.117 | 0.907 | -0.02 |
| CC Mid Anterior | 0.000377±1.012E-04 | 0.000369±9.56E-05 | -0.735 | 0.462 | -0.09 |
| CC Anterior | 0.000574±8.74E-05 | 0.000568±8.52E-05 | -0.849 | 0.396 | -0.07 |
| Amygdala | 0.00116±9.78E-05 | 0.00117±1.023E-04 | 1.184 | 0.237 | 0.07 |
| Hippocampus | 0.00267±0.000205 | 0.00266±0.000213 | 0.201 | 0.841 | -0.01 |
| CB GM | 0.0392±0.00304 | 0.0392±0.00313 | 0.089 | 0.929 | 0 |
| CB WM | 0.00850±0.000768 | 0.00857±0.000849 | 0.673 | 0.501 | 0.09 |
| Caudate | 0.00267±0.000291 | 0.00269±0.000271 | 0.407 | 0.684 | 0.06 |
| Putamen | 0.00352±0.000329 | 0.00355±0.000346 | 0.535 | 0.593 | 0.08 |
| Pallidum | 0.00129±0.000133 | 0.00129±0.000117 | 0.391 | 0.696 | 0.01 |
| Accumbens | 0.000443±4.78E-05 | 0.000452±5.47E-05 | 1.267 | 0.205 | 0.17 |
| Amyg-Hipp | 0.00383±0.000267 | 0.00383±0.000281 | 0.588 | 0.556 | 0.02 |
| Thalamus Proper | 0.00496±0.000327 | 0.00493±0.000316 | -1.164 | 0.244 | -0.1 |
| Lateral Ventricle | 0.00371±0.00232 | 0.00360±0.00174 | -0.581 | 0.562 | -0.06 |
| Inf Lateral Vent | 0.000248±9.067E-05 | 0.000253±8.281E-05 | 1.097 | 0.273 | 0.06 |
| Third Ventricle | 0.000512±0.000182 | 0.000498±0.000131 | -0.627 | 0.531 | -0.11 |
| Fourth Ventricle | 0.0114±0.000327 | 0.00112±0.000297 | -0.281 | 0.779 | -0.06 |

*Footnotes: Regions in bold indicate a significant difference between those with versus those without ARFID symptoms at the uncorrected level.  ^a^ T and* d *values reported as No ARFID S. – ARFID S.; ^b^ Marked regions are not corrected for head size; * - p(FDR)<0.05; ** - p(FDR)<0.01; *** - p(FDR)<0.001.*

*[Abbreviations: ACC – Anterior Cingulate Cortex; Amyg-Hipp – Amygdalar-Hippocampal Boundary; ARFID – Avoidant/Restrictive Food Intake Disorder; CB – Cerebellum; CC – Cingulate Cortex; CSF – Cerebrospinal Fluid; DC – Diencephalon; eTIV - estimated total Intracranial Volume; M – Mean; MF – Middle Frontal; GM – Gray Matter; OFC – Orbitofrontal Cortex; S. – Symptoms; SD – Standard Deviation; STS – Superior Temporal Sulcus; Vent – Ventricle; WM – White Matter]*

| **Table S3. Exploratory cortical thickness, surface area and volume of those with (n=121) vs. without (n=1,856) ARFID symptoms post-BMI correction** | | | | | | |
| --- | --- | --- | --- | --- | --- | --- |
| **Region - Thickness** | **M±SD ARFID (mm^3^)** | **M±SD NO ARFID S. (mm^3^)** | **t (Tukey)^a^** | **p** | ***d* ^a^** |  |
| MF Caudal | 2.74±0.14 | 2.72±0.13 | -1.918 | 0.0553 | -0.13 |  |
| **MF Rostral** | **2.66±0.12** | **2.64±0.11** | **-2.234** | **0.0256** | -0.19 |  |
| **Banks STS** | **2.83±0.15** | **2.81±0.15** | **-2.201** | **0.0278** | -0.15 |  |
| Entorhinal | 3.25±0.32 | 3.25±0.31 | -0.294 | 0.769 | 0.02 |  |
| Fusiform | 2.93±0.11 | 2.92±0.11 | -1.374 | 0.169 | -0.06 |  |
| Inferior Parietal | 2.74±0.12 | 2.74±0.12 | -2.211 | 0.0272 | -0.08 |  |
| Inferior Temporal | 2.78±0.15 | 2.77±0.13 | -1.61 | 0.108 | -0.09 |  |
| Lateral Occipital | 2.25±0.10 | 2.25±0.11 | -0.855 | 0.393 | 0 |  |
| Lingual | 2.18±0.13 | 2.17±0.13 | -0.527 | 0.598 | -0.05 |  |
| **Middle Temporal** | **2.98±0.17** | **2.95±0.14** | **-2.462** | **0.0139** | -0.19 |  |
| Parahippocampal | 3.00±0.20 | 3.01±0.22 | 0.108 | 0.914 | 0.07 |  |
| Paracentral | 2.71±0.12 | 2.70±0.12 | -1.309 | 0.191 | -0.09 |  |
| Precentral | 2.62±0.11 | 2.61±0.11 | -1.407 | 0.16 | -0.07 |  |
| Postcentral | 2.20±0.11 | 2.22±0.11 | -0.111 | 0.911 | 0.1 |  |
| **Precuneus** | **2.79±0.09** | **2.78±0.10** | **-1.98** | **0.0479** | -0.12 |  |
| Cuneus | 2.08±0.14 | 2.08±0.15 | -0.04 | 0.968 | 0 |  |
| **Superior Frontal** | **3.02±0.13** | **3.00±0.12** | **-3.329** | **0.000889*** | **-0.28** |  |
| Superior Parietal | 2.42±0.11 | 2.42±0.11 | -1.55 | 0.121 | -0.01 |  |
| **Superior Temporal** | **2.94±0.15** | **2.92±0.14** | **-2.173** | **0.0299** | -0.13 |  |
| **Frontal** | **2.80±0.10** | **2.78±0.09** | **-2.743** | **0.00615** | -0.21 |  |
| **Temporal** | **2.92±0.12** | **2.91±0.11** | **-2.131** | **0.0332** | -0.13 |  |
| Parietal | 2.58±0.09 | 2.58±0.09 | -1.909 | 0.0565 | -0.04 |  |
| Occipital | 2.14±0.10 | 2.14±0.10 | -0.85 | 0.395 | -0.04 |  |
| Parsopercularis | 2.84±0.13 | 2.83±0.12 | -1.476 | 0.14 | -0.07 |  |
| Parsorbitalis | 2.93±0.17 | 2.91±0.17 | -1.682 | 0.0928 | -0.15 |  |
| Parstriangularis | 2.71±0.14 | 2.71±0.12 | -0.976 | 0.329 | -0.07 |  |
| Pericalcarine | 1.68±0.15 | 1.67±0.16 | -0.49 | 0.624 | -0.05 |  |
| Supramarginal | 2.80±0.11 | 2.80±0.11 | -1.606 | 0.108 | -0.07 |  |
| ACC Caudal | 2.87±0.20 | 2.88±0.20 | 0.653 | 0.514 | 0.05 |  |
| ACC Rostral | 3.30±0.17 | 3.30±0.18 | -0.387 | 0.699 | -0.01 |  |
| Cingulate | 2.89±0.11 | 2.89±0.11 | -0.124 | 0.901 | 0.03 |  |
| Insula | 3.25±0.14 | 3.24±0.13 | -0.19 | 0.85 | -0.02 |  |
| OFC Lateral | 2.93±0.12 | 2.92±0.13 | -0.97 | 0.332 | -0.11 |  |
| **OFC Medial** | **2.78±0.14** | **2.75±0.15** | **-2.111** | **0.0349** | -0.17 |  |
| Transverse Temporal | 2.70±0.18 | 2.68±0.18 | -1.268 | 0.205 | -0.13 |  |
| Isthmus Cingulate | 2.77±0.18 | 2.77±0.16 | -0.654 | 0.513 | 0 |  |
| Temporal Pole | 3.39±0.40 | 3.40±0.37 | 0.017 | 0.986 | 0 |  |
| Frontal Pole | 2.85±0.35 | 2.85±0.34 | 0.168 | 0.867 | 0 |  |
| **^b^ Region - Surface Area** | **M±SD ARFID (mm^3^)** | **M±SD NO ARFID S. (mm^3^)** | **t (Tukey)^a^** | **p** | ***d* ^a^** |  |
| MF Caudal | 0.00158±0.000197 | 0.00158±0.000193 | 0.515 | 0.607 | 0.015 |  |
| MF Rostral | 0.00417±0.000476 | 0.000415±0.000447 | -0.555 | 0.579 | -0.062 |  |
| Banks STS | 0.000673±8.06E-05 | 0.000671±8.03E-05 | 0.199 | 0.843 | -0.028 |  |
| Entorhinal | 0.000278±4.86E-05 | 0.000286±5.07E-05 | 1.581 | 0.114 | 0.17 |  |
| Fusiform | 0.00214±0.000199 | 0.00215±0.000187 | 0.15 | 0.88 | 0.05 |  |
| Inferior Parietal | 0.00370±0.000419 | 0.00371±0.000385 | 0.503 | 0.615 | 0.01 |  |
| Inferior Temporal | 0.00234±0.000299 | 0.00234±0.000252 | 0.394 | 0.694 | -0.01 |  |
| Lateral Occipital | 0.00397±0.000362 | 0.00392±0.000400 | -1.003 | 0.316 | -0.13 |  |
| Lingual | 0.00225±0.000256 | 0.00221±0.000260 | -1.314 | 0.189 | -0.14 |  |
| Middle Temporal | 0.00233±0.000265 | 0.00231±0.000217 | -0.044 | 0.965 | -0.08 |  |
| Parahippocampal | 0.000443±4.53E-05 | 0.000445±4.26E-05 | 1.118 | 0.264 | 0.05 |  |
| Paracentral | 0.000999±8.56E-05 | 0.00100±9.23E-05 | 0.426 | 0.67 | 0.03 |  |
| Precentral | 0.00333±0.000241 | 0.00335±0.000255 | 1.211 | 0.226 | 0.08 |  |
| Postcentral | 0.00289±0.000257 | 0.00292±0.000242 | 1.752 | 0.08 | 0.12 |  |
| Precuneus | 0.00286±0.000263 | 0.00285±0.000256 | -0.396 | 0.692 | -0.02 |  |
| Cuneus | 0.00112±0.000134 | 0.00112±0.000139 | 0.591 | 0.555 | 0.02 |  |
| Superior Frontal | 0.000513±0.000448 | 0.00514±0.000433 | 0.202 | 0.84 | 0.04 |  |
| Superior Parietal | 0.00407±0.000409 | 0.00408±0.000404 | 0.334 | 0.739 | 0.01 |  |
| Superior Temporal | 0.00258±0.000244 | 0.00260±0.000204 | 1.368 | 0.172 | 0.12 |  |
| ^b^ Frontal | 31956.76±3538.78 | 32178.46±3601.77 | 0.592 | 0.554 | 0.06 |  |
| ^b^ Temporal | 17130.63±1916.43 | 17284.54±1872.55 | 1.099 | 0.272 | 0.08 |  |
| ^b^ Parietal | 24579.91±2710.78 | 24853.80±2781.48 | 1.234 | 0.217 | 0.1 |  |
| ^b^ Occipital | 12631.51±1311.32 | 12565.65±1481.43 | -0.503 | 0.615 | -0.04 |  |
| Parsopercularis | 0.00105±0.000124 | 0.00104±0.000123 | -0.405 | 0.685 | -0.03 |  |
| Parsorbitalis | 0.000539±5.73E-05 | 0.000537±5.77E-05 | 0.125 | 0.901 | -0.03 |  |
| Parstriangularis | 0.00101±0.000116 | 0.00100±0.000123 | -0.201 | 0.84 | -0.02 |  |
| Pericalcarine | 0.00103±0.000143 | 0.00102±0.000161 | -0.404 | 0.686 | -0.04 |  |
| Supramarginal | 0.00273±0.000317 | 0.00278±0.000309 | 1.743 | 0.0815 | 0.16 |  |
| ACC Caudal | 0.000447±6.31E-05 | 0.000450±6.29E-05 | -0.139 | 0.889 | 0.05 |  |
| ACC Rostral | 0.000488±7.52E-05 | 0.000495±7.20E-05 | 0.252 | 0.801 | 0.1 |  |
| Cingulate | 0.00249±0.000211 | 0.00248±0.000203 | -0.853 | 0.394 | -0.02 |  |
| Insula | 0.00157±0.000142 | 0.00158±0.000133 | -0.195 | 0.845 | 0.09 |  |
| OFC Lateral | 0.00181±0.000198 | 0.00182±0.000200 | 0.255 | 0.799 | 0.07 |  |
| OFC Medial | 0.00130±0.000132 | 0.00130±0.000129 | -0.312 | 0.755 | 0.02 |  |
| **Transverse Temporal** | **0.000261±3.04E-05** | **0.000268±3.01E-05** | **2.075** | **0.0381** | 0.43 |  |
| **Isthmus Cingulate** | **0.000703±6.61E-05** | **0.000691±7.33E-05** | **-2.013** | **0.0443** | -0.16 |  |
| Temporal Pole | 0.000298±3.59E-05 | 0.000296±3.33E-05 | -0.565 | 0.572 | -0.07 |  |
| Frontal Pole | 0.000209±1.99E-05 | 0.000208±2.09E-05 | -0.553 | 0.58 | -0.04 |  |
| **^b^ Region - Volume** | **M±SD ARFID S. (mm^3^)** | **M±NO ARFID S. (mm^3^)** | **t (Tukey)^a^** | **p** | ***d* ^a^** |  |
| ^b^ eTIV | 1512370.0±125440.8 | 1522073.0±139557.5 | 0.543 | 0.587 | 0.07 |  |
| ^b^ GM | 289907.9±28027.3 | 290990.2±27982.1 | -0.071 | 0.944 | 0.04 |  |
| ^b^ GM Cortex | 579815.8±56054.6 | 581980.3±55964.3 | -0.071 | 0.944 | 0.04 |  |
| ^b^ GM Subcort | 60276.3±4170.5 | 60731.5±4610.4 | 0.262 | 0.793 | 0.1 |  |
| ^b^ GM Total | 759353.0±65780.1 | 762641.4±66296.2 | 0.026 | 0.979 | 0.05 |  |
| ^b^ WM | 210866.8±24107.3 | 211967.7±25362.2 | 0.041 | 0.967 | 0.04 |  |
| ^b^ WM Total | 421733.5±48214.6 | 423935.4±50724.4 | 0.041 | 0.967 | 0.04 |  |
| Brainstem | 0.0123±0.000785 | 0.0123±0.000939 | -0.604 | 0.546 | -0.01 |  |
| CSF | 0.000600±0.000129 | 0.000600±0.000120 | -0.318 | 0.751 | 0 |  |
| Ventral DC | 0.00269±0.000191 | 0.00269±0.000190 | -0.172 | 0.863 | 0.05 |  |
| Vessel | 2.02E-05±8.52E-06 | 2.11E-05±1.15E-05 | 0.184 | 0.854 | 0.08 |  |
| Choroid Plexus | 0.000274±6.71E-05 | 0.000274±6.66E-05 | -0.269 | 0.788 | 0 |  |
| ^b^ **Mean Thickness** | **2.69±0.081** | **2.68±0.079** | **-2.397** | **0.0166** | -0.13 |  |
| CC Posterior | 0.000562±7.66E-05 | 0.000559±8.54E-05 | -0.563 | 0.573 | -0.03 |  |
| CC Mid Posterior | 0.000324±4.98E-05 | 0.000325±5.69E-05 | -0.302 | 0.763 | 0.02 |  |
| CC Central | 0.000365±9.20E-05 | 0.000363±8.77E-05 | -0.26 | 0.795 | -0.02 |  |
| CC Mid Anterior | 0.000377±1.012E-04 | 0.000369±9.56E-05 | -1.224 | 0.221 | -0.09 |  |
| CC Anterior | 0.000574±8.74E-05 | 0.000568±8.52E-05 | -0.81 | 0.418 | -0.07 |  |
| Amygdala | 0.00116±9.78E-05 | 0.00117±1.023E-04 | 0.389 | 0.697 | 0.07 |  |
| Hippocampus | 0.00267±0.000205 | 0.00266±0.000213 | -0.225 | 0.822 | -0.01 |  |
| CB GM | 0.0392±0.00304 | 0.0392±0.00313 | 0.022 | 0.982 | 0 |  |
| CB WM | 0.00850±0.000768 | 0.00857±0.000849 | -1.001 | 0.317 | 0.09 |  |
| Caudate | 0.00267±0.000291 | 0.00269±0.000271 | 0.291 | 0.771 | 0.06 |  |
| Putamen | 0.00352±0.000329 | 0.00355±0.000346 | 0.182 | 0.855 | 0.08 |  |
| Pallidum | 0.00129±0.000133 | 0.00129±0.000117 | -0.092 | 0.927 | 0.01 |  |
| Accumbens | 0.000443±4.78E-05 | 0.000452±5.47E-05 | 1.243 | 0.214 | 0.17 |  |
| Amyg-Hipp | 0.00383±0.000267 | 0.00383±0.000281 | -0.027 | 0.978 | 0.02 |  |
| Thalamus Proper | 0.00496±0.000327 | 0.00493±0.000316 | -1.626 | 0.104 | -0.1 |  |
| Lateral Ventricle | 0.00371±0.00232 | 0.00360±0.00174 | -0.708 | 0.479 | -0.06 |  |
| Inf Lateral Vent | 0.000248±9.067E-05 | 0.000253±8.281E-05 | 0.922 | 0.357 | 0.06 |  |
| Third Ventricle | 0.000512±0.000182 | 0.000498±0.000131 | -1.061 | 0.289 | -0.11 |  |
| Fourth Ventricle | 0.0114±0.000327 | 0.00112±0.000297 | -0.3 | 0.764 | -0.06 |  |

*Footnotes: Regions in bold indicate a significant difference between those with versus those without ARFID symptoms at the uncorrected level.* ***^a^*** *T and* d *values reported as No ARFID S. – ARFID S.; ^b^ Marked regions are not corrected for head size; * - p(FDR)<0.05; ** - p(FDR)<0.01; *** - p(FDR)<0.001.*

*[Abbreviations: ACC – Anterior Cingulate Cortex; Amyg-Hipp – Amygdalar-Hippocampal Boundary; ARFID – Avoidant/Restrictive Food Intake Disorder; CB – Cerebellum; CC – Cingulate Cortex; CSF – Cerebrospinal Fluid; DC – Diencephalon; eTIV - estimated total Intracranial Volume; M – Mean; MF – Middle Frontal; GM – Gray Matter; OFC – Orbitofrontal Cortex; S. – Symptoms; SD – Standard Deviation; STS – Superior Temporal Sulcus; Vent – Ventricle; WM – White Matter]*
